# Supplementary figures and images for: A non-canonical role of somatic Cyclin D/CYD-1 in oogenesis and in maintenance of reproductive fidelity, dependent on the FOXO/DAF-16 activation state
Source: PLoS Genet. 2024 Nov 15;20(11):e1011453. doi: 10.1371/journal.pgen.1011453 (PMC11602045; doi:10.1371/journal.pgen.1011453)

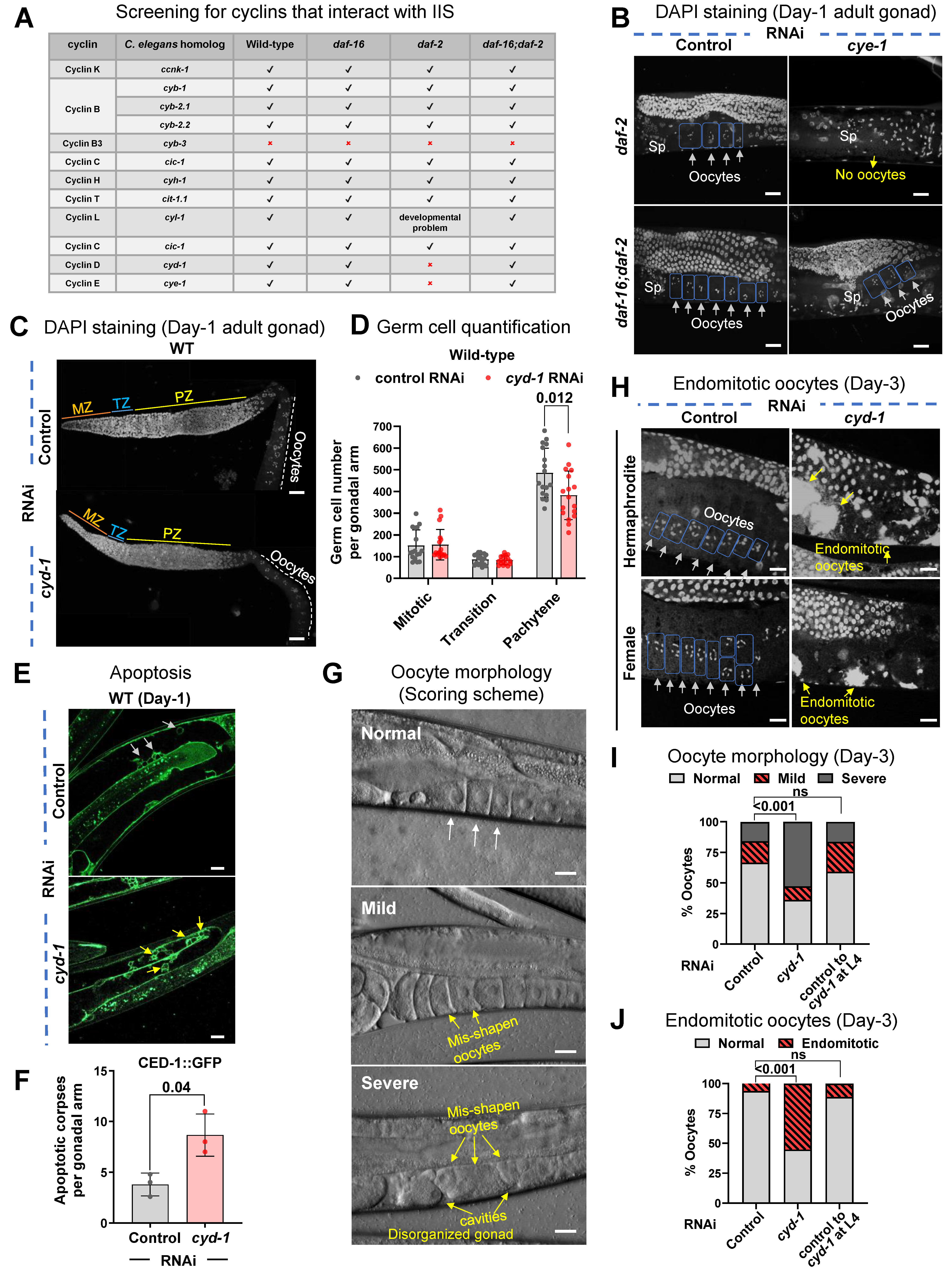

Supplement: S1 Fig — (A) RNAi screen for cyclins that may interact with the IIS. Twelve cyclin genes were knocked down in WT, daf-16(mgdf50), daf-2(e1370) and daf-16(mgdf50);daf-2(e1370) using RNAi. The tick mark indicates fertile worms while the cross mark indicates sterility when grown on respective RNAi from L1 onwards. (B) Representative fluorescent images of DAPI-stained germ line of daf-2(e1370) and daf-16(mgdf50);daf-2(e1370) worms grown on control or cye-1 RNAi. Oocytes are boxed for clarity. White arrows point towards oocytes while yellow shows the absence of oocytes. Sp denotes sperms. (C,D) Representative images of DAPI-stained dissected gonadal arm of WT worms (day-1 adult) grown on control or cyd-1 RNAi. Mitotic zone (MT), transition Zone (TZ), and pachytene zone (PZ) germ cells are marked with a solid line and white dashed line mark oocytes (C). Quantification of germ cells in each zone (D). Each point represents the number of germ cells in the respective zones (n = 17). Unpaired t-test with Welch’s correction. (E,F) Representative images showing apoptotic cells (marked by arrows) in the gonadal arm of ced-1::gfp worms (day-1 adult) grown on control or cyd-1 RNAi (E). Quantification for apoptotic cells per gonadal arm (F) The average of three biological replicates is shown (n ≥ 15 for each replicate). Unpaired t-test with Welch’s correction. (G) Oocyte quality score based on morphology. The quality was categorized as normal, mild or severe based on the presence of cavities, shape and organization of oocytes. Normal = No cavities/not misshapen/not disorganized, mild = either with cavities/ are misshapen/disorganized (≤ 2 instances per worm), severe = either with cavities/ are misshapen/disorganized (≥ 3 instances per worm). (H) Representative DAPI-stained gonads of WT hermaphrodites (day-3 adult) and fog-2(q71) females grown on control or cyd-1 RNAi at 25°C. Oocytes are boxed for clarity. White arrows point towards oocytes while yellow arrows point towards endomitotic oocyt [file pgen.1011453.s001.tif]

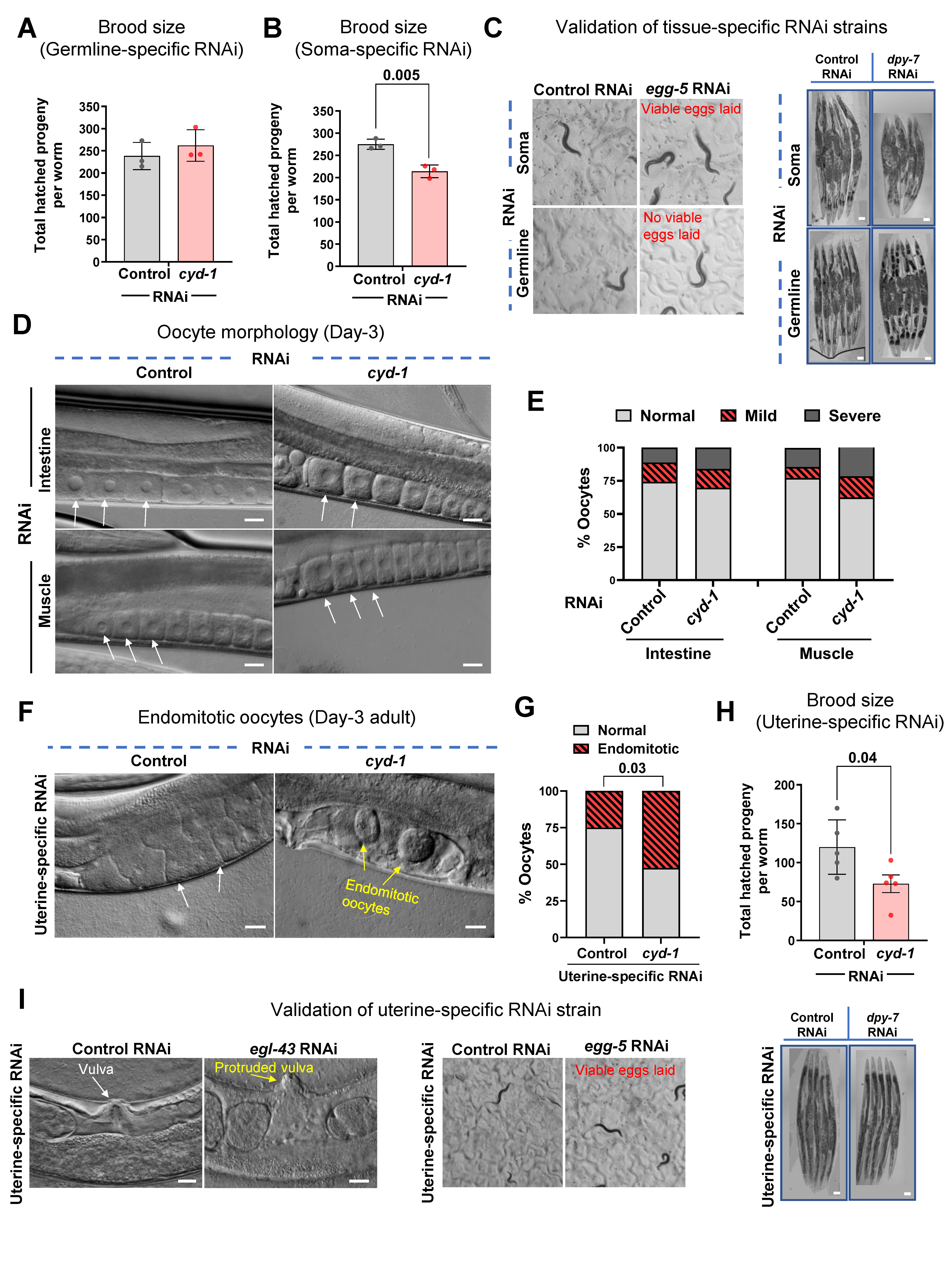

Supplement: S2 Fig — (A) The total number of hatched progenies in rde-1(mkc36);sun-1p::rde-1 worms (germline-specific RNAi) grown on control or cyd-1 RNAi. Average of three biological repeats. Unpaired t-test with Welch’s correction. (B) The total number of hatched progenies in ppw-1(pk1425) worms (soma-specific RNAi) grown on control or cyd-1 RNAi. Average of three biological repeats. Unpaired t-test with Welch’s correction. (C) Representative images of ppw-1(pk1425) (soma-specific RNAi), or rde-1(mkc36);sun-1p::rde-1 (germline-specific RNAi) worms (day-1 adult) grown on control, egg-5 or dpy-7 RNAi. KD of egg-5 led to non-viable progeny in the germline-specific RNAi strain but not in the soma-specific RNAi strain, while KD of dpy-7 led to dumpy phenotype in the soma-specific RNAi strain but not in the germline-specific RNAi strain. Scale bar 100 μm. (D,E) DIC images showing oocyte morphology of rrf-3(pk1426);rde-1(ne219);nhx-2p::rde-1 (intestine-specific RNAi) or rrf-3(pk1426);rde-1(ne219);hlh-1p::rde-1 (muscle-specific RNAi) worms (day-3 adult) grown on control or cyd-1 RNAi. White arrows mark oocytes. Scale bar 20 μm (D). Oocyte quality score (based on morphology as shown in S1G Fig) (E). Combined data from three biological replicates (n ≥ 34) is plotted. Chi-square analysis was used to compare between groups. (F,G) DIC images showing the presence of unfertilized oocytes/endomitotic oocytes (emos) in the uterus of rrf-3(pk1426);rde-1(ne219);fos-1ap::rde-1(genomic) (uterine tissue-specific RNAi) worms (day-3 adult) grown on control or cyd-1 RNAi. White arrows mark unfertilized oocytes in the uterus while yellow arrows mark endomitotic oocytes (emos). Scale bar 20 μm (F). Quantification for endomitotic oocytes (G) Combined data from three biological replicates (n ≥ 24) is plotted. Chi-square analysis was used to compare between groups. (H) The total number of hatched progenies in rrf-3(pk1426);rde-1(ne219);fos-1ap::rde-1(genomic) (uterine tissue-specific RNAi) worms grown on control o [file pgen.1011453.s002.tif]

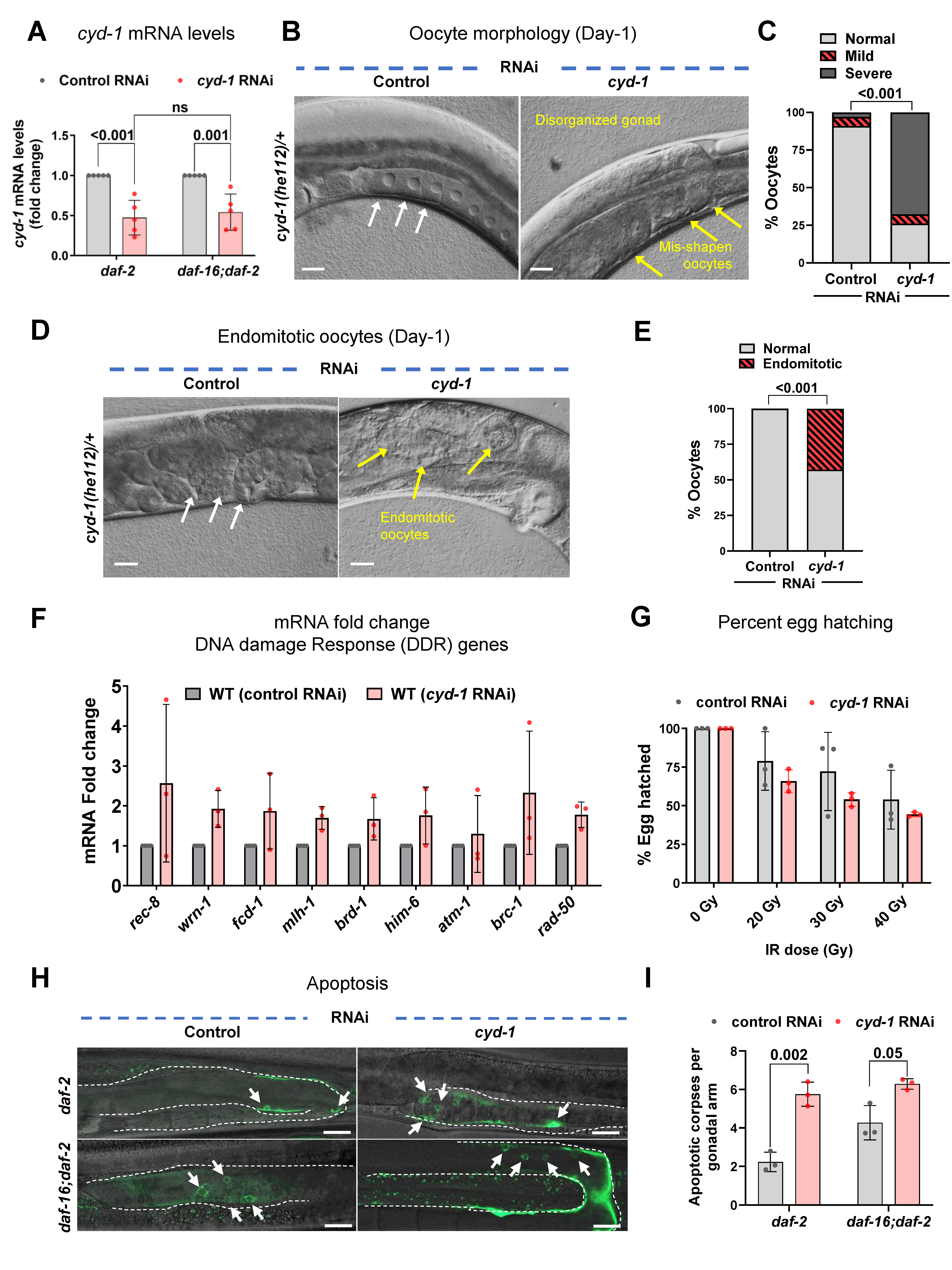

Supplement: S3 Fig — (A) RT-PCR analysis showing knockdown efficiency of cyd-1 RNAi in daf-2(e1370) and daf-16(mgdf50);daf-2(e1370). Expression levels were normalized to actin. Average of 5 biological replicates are shown. Two-way ANOVA-Tukey’s multiple comparisons test. (B,C) DIC images showing oocyte morphology of cyd-1(he112)/+ (day-1 adult) worms grown on control or cyd-1 RNAi. White arrows mark normal oocytes whereas yellow arrows mark oocytes with cavities or those that are misshapen or disorganized, indicative of poor quality (B). Oocyte quality score (based on morphology as shown in S1G Fig) (C). Combined data from three biological replicates (n ≥ 33) is plotted. Chi-square analysis was used to compare between groups. (D,E) DIC images showing eggs or endomitotic oocytes (emos) in cyd-1(he112)/+ (day-1 adult) worms grown on control or cyd-1 RNAi. White arrows mark normal eggs in the uterus whereas yellow arrows mark emos (D). Quantification for endomitotic oocytes. (E) Combined data from three biological replicates (n ≥ 20) is plotted. Chi-square analysis was performed to compare between groups. (F) Quantitative RT-PCR analysis for DNA damage response (DDR) genes in WT late-L4 staged worms grown on control or cyd-1 RNAi. Expression levels were normalized to actin. Average of three biological replicates are shown. Unpaired t-test with Welch’s correction. (G) The percentage of eggs hatched in control or cyd-1 RNAi fed WT L4-staged worms exposed to different doses of IR (0, 20, 30, 40 Gy). The average of three biological replicates is shown (n≥20). Unpaired t-test with Welch’s correction (H,I) Representative fluorescent and DIC merged images showing apoptotic cells (arrows) in the gonadal arm of daf-2(e1370);ced-1::gfp and daf-16(mgdf50);daf-2(e1370);ced-1::gfp (day-1 adult) worms grown on control or cyd-1 RNAi. Arrows mark apoptotic corpses. (H). Quantification for apoptotic corpses per gonadal arm (I). An average of three biological replicates are shown (n ≥ 17 for each replicate) [file pgen.1011453.s003.tif]

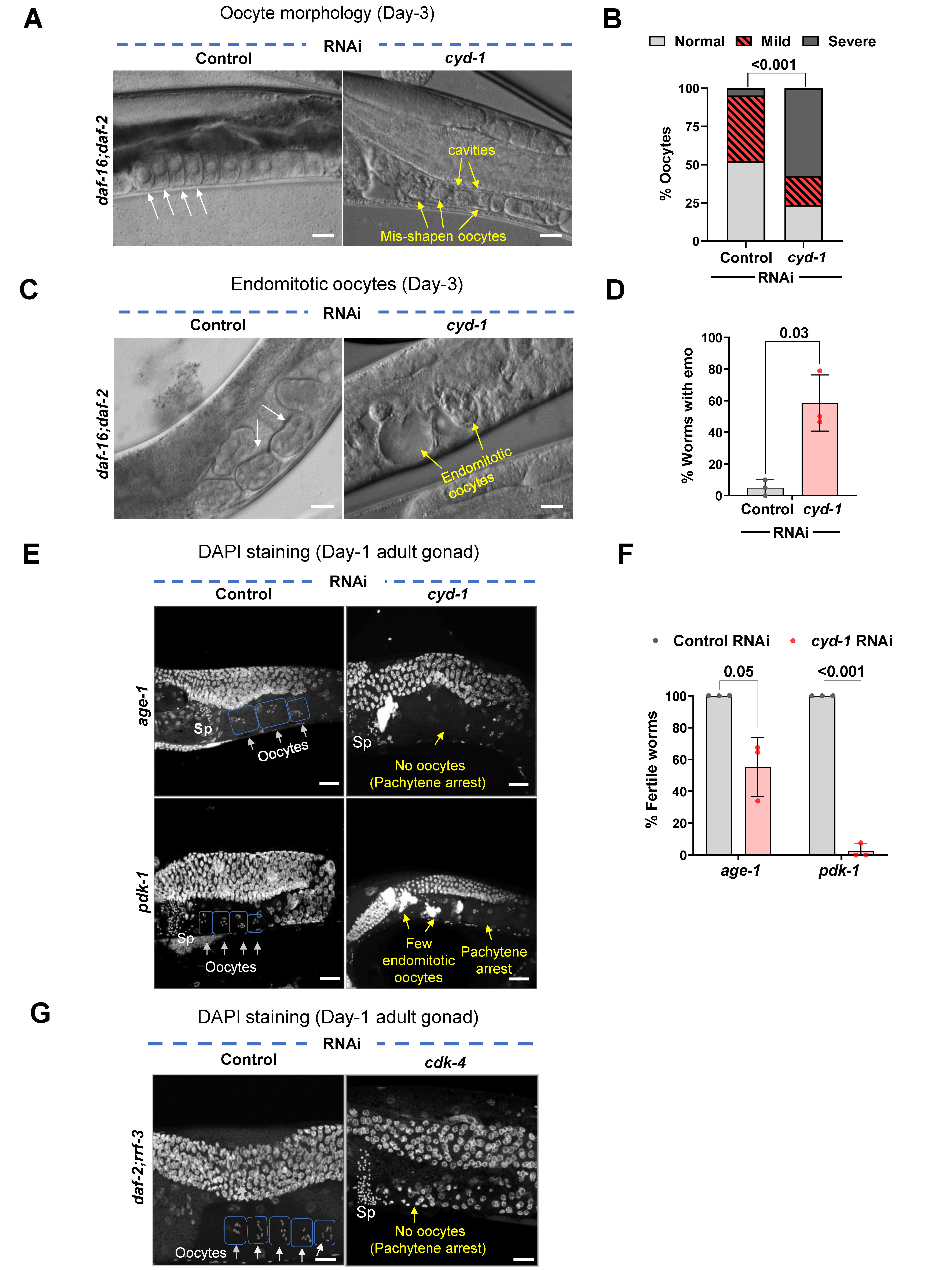

Supplement: S4 Fig — (A,B) DIC images showing oocyte morphology of daf-16(mgdf50);daf-2(e1370) (day-3 adult) worms grown on control or cyd-1 RNAi. White arrows mark normal oocytes whereas yellow arrows mark oocytes with cavities or those that are misshapen or disorganized, indicative of poor quality (A). Oocyte quality score (based on morphology as shown in S1G Fig) (B). Combined data from three biological replicates (n ≥ 32) is plotted. Chi-square analysis was used to compare between groups. (C,D) DIC images showing eggs or endomitotic oocytes (emos) in daf-16(mgdf50);daf-2(e1370) (day-3 adult) worms grown on control or cyd-1 RNAi. White arrows mark normal eggs in the uterus whereas yellow arrows mark emos (C). Quantification for endomitotic oocytes (D). Average of three biological replicates (n ≥ 20 for each replicate). Unpaired t-test with Welch’s correction. (E,F) Representative fluorescent images of DAPI stained gonads of age-1(hx546) and pdk-1(sa680) (day-1 adult) worms grown on control or cyd-1 RNAi. Oocytes are boxed for clarity. White arrows point towards oocytes while yellow shows the absence of oocytes. Sp denotes sperms (E). The percentage of fertile worms (F). Average of three biological replicates (n ≥ 30 for each replicate). Unpaired t-test with Welch’s correction. (G) Representative DAPI-stained gonads of rrf-3(pk1426);daf-2(e1370) worms (day-1 adult) grown on control or cdk-4 RNAi. Oocytes are boxed for clarity. White arrows point towards oocytes while yellow shows the absence of oocytes. Scale bars:20 μm. Error bars are s.d. Experiments were performed at 20°C. Source data are provided in S1 Table. (TIF) [file pgen.1011453.s004.tif]

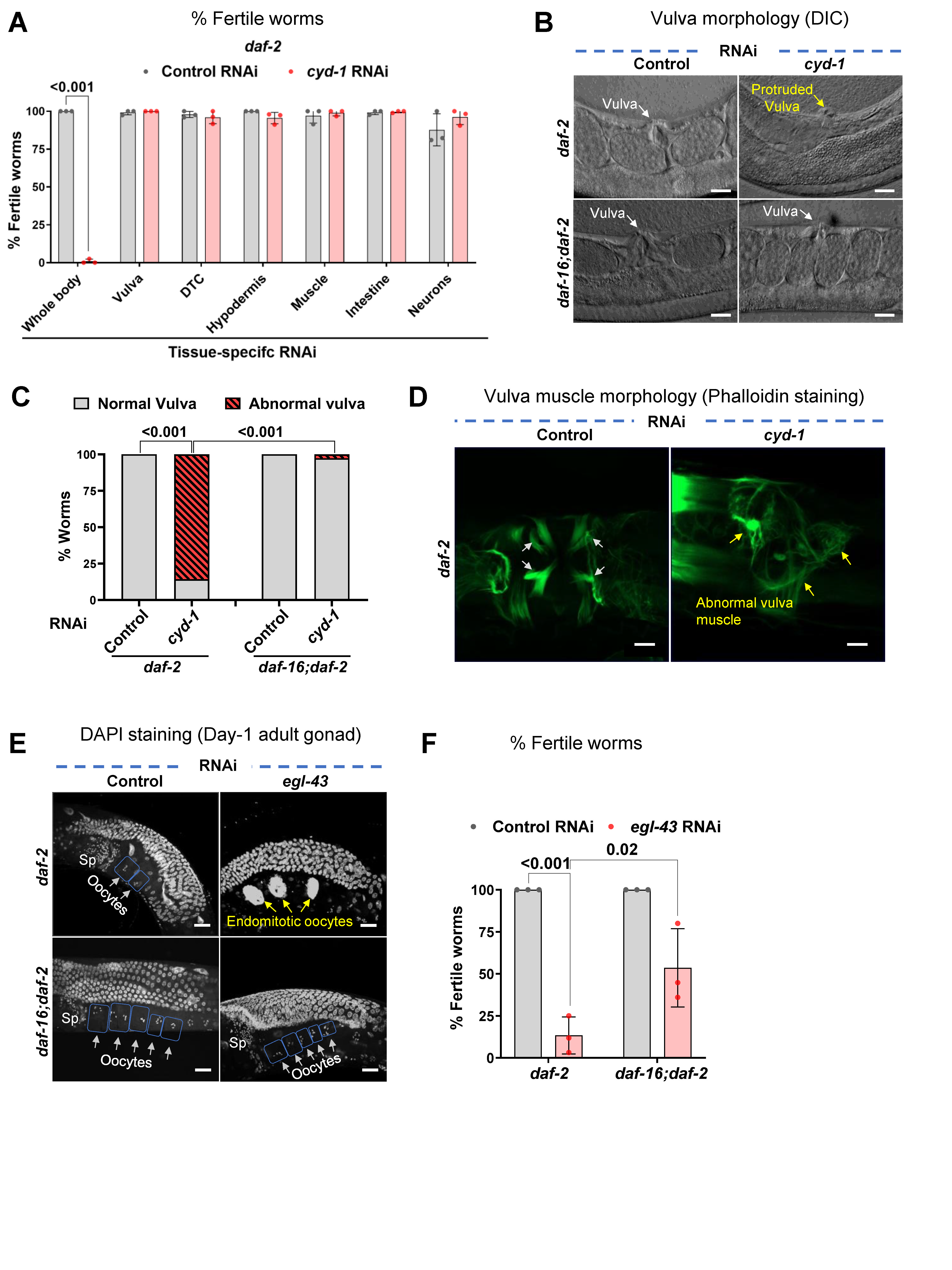

Supplement: S5 Fig — (A) The percentage of fertile worms upon tissue-specific cyd-1 KD in daf-2(e1370). The following promoters were used to drive the tissue-specific expression of rde-1 (Vulva: lin-31p; DTC: lag-2p; Hypodermis: lin-26p; muscle: hlh-1p; intestine: nhx-2p) in a rde-1(ne219) mutant. For neuron-specific RNAi, unc-119p was used to drive the expression of sid-1 cDNA in the neurons of sid-1(pk3321) mutant. Average of three biological replicates (n ≥ 25 per condition for each experiment). Unpaired t-test with Welch’s correction. (B,C) DIC images of gonads of daf-2(e1370) and daf-16(mgdf50);daf-2(e1370) worms (day-1 adult) on control or cyd-1 RNAi. White arrows point towards normal vulva while yellow points towards abnormal vulva (protruded). Scale bar 100 μm (B). Combined data from three biological replicates (n ≥ 22) is plotted. Chi-square analysis was used to compare between groups. (D) Representative fluorescent images of Phalloidin-stained (that marks the F-actin) gonads of daf-2(e1370) worms (day-1 adult) grown on control or cyd-1 RNAi. White arrows show normal vulva muscle structure. Yellow arrows show defective vulva muscles. Scale bar 10 μm. (E,F) Representative fluorescent images of DAPI-stained gonads of daf-2(e1370) and daf-16(mgdf50);daf-2(e1370) (day-1 adult) worms grown on control or egl-43 RNAi. Oocytes are boxed for clarity. White arrows point towards oocytes while yellow shows endomitotic oocytes. Sp denotes sperms. Scale bar 20 μm (E). The percentage of fertile worms (F). Average of three biological replicates (n ≥ 25 per condition for each experiment). Two-way ANOVA-Tukey’s multiple comparisons test. Error bars are s.d. Experiments were performed at 20°C. Source data are provided in S1 Table. (TIF) [file pgen.1011453.s005.tif]

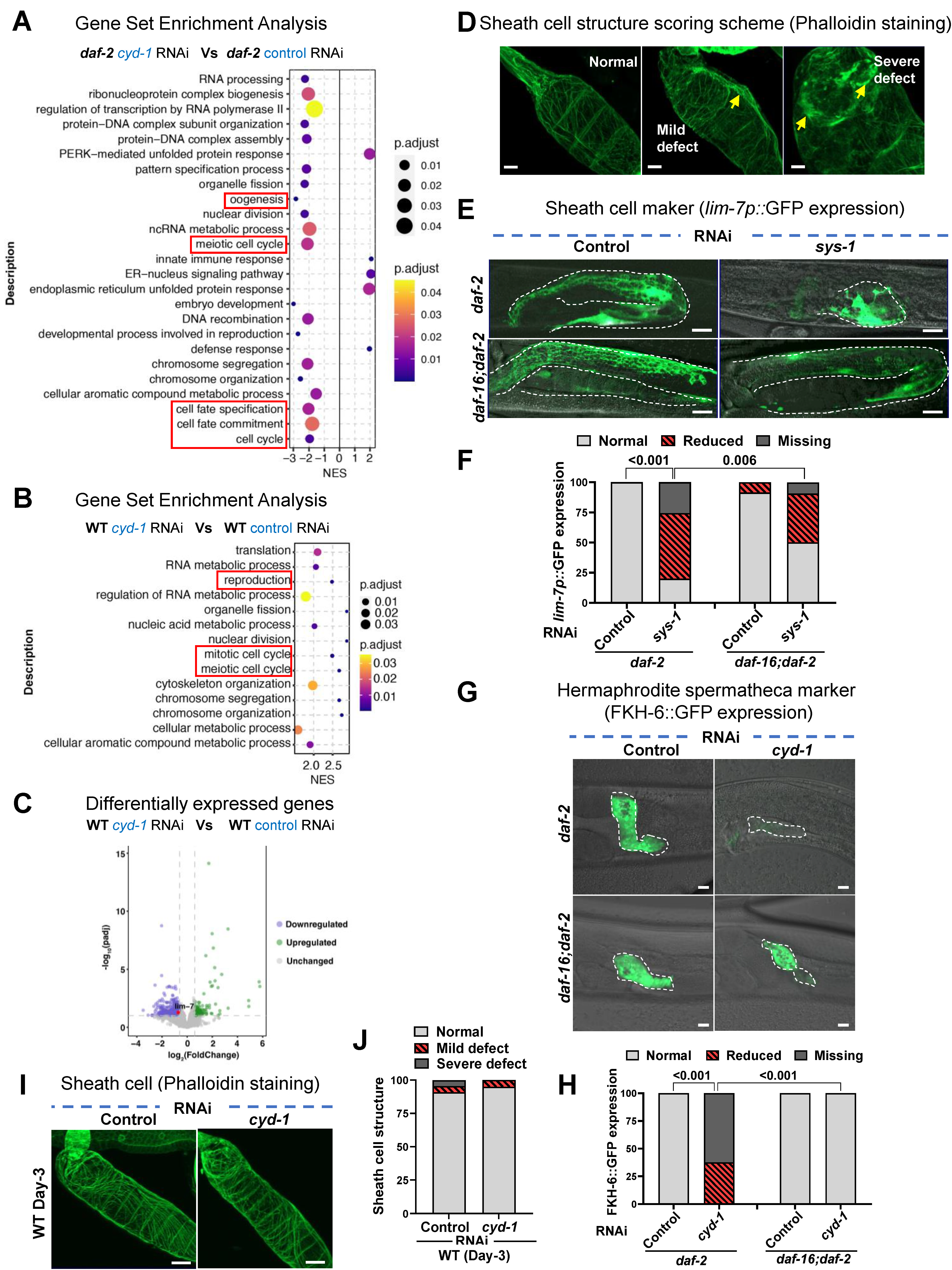

Supplement: S6 Fig — (A-B) Gene set enrichment analysis of differentially expressed genes in (A) daf-2(e1370);rrf-3(pk1426);rde-1(ne219);unc-62p::rde-1(genomic) or (B) rrf-3(pk1426);rde-1(ne219);unc-62p::rde-1(genomic) upon cyd-1 KD as compared to control RNAi. (C) Volcano plot showing the magnitude [log2(FC)] and significance [−log10(P value)] of the genes that are differentially expressed in L4 stage of rrf-3(pk1426);rde-1(ne219);unc-62p::rde-1(genomic) worms, grown on control or cyd-1 RNAi. (D) Sheath cell structure scoring scheme. Representative fluorescent images of Phalloidin-stained (that marks the F-actin) gonads of worms (day-1 adult). The quality was categorized as normal, mild or severe based on the structure and organization of actin filaments. Normal = No loosened or disorganized structure, mild = either loosened or disorganized actin filaments, severe = highly loosened or disorganized actin filaments. Yellow arrows point towards the defect. (E,F) Representative fluorescent and DIC merged images of gonads showing lim-7p::GFP (that marks the sheath cells) expression in daf-2(e1370) and daf-16(mgdf50);daf-2(e1370) worms (day-1 adult) grown on control and sys-1 RNAi. The gonadal arm is outlined for clarity. Scale bar 20 μm (E). Quantification of the normal, reduced or missing expression of lim-7p::GFP (F). Combined data from three biological replicates (n ≥ 32) is plotted. Chi-square analysis was used to compare between groups. (G,H) Representative fluorescent and DIC merged images of gonads showing FKH-6::GFP (that marks the hermaphrodite spermatheca) expression in daf-2(e1370) and daf-16(mgdf50);daf-2(e1370) worms (day-1 adult) grown on control and cyd-1 RNAi. Spermatheca is outlined for clarity. Scale bar 20 μm (G). Quantification of the normal or missing expression of FKH-6::GFP (H). Combined data from three biological replicates (n ≥ 24) is plotted. Chi-square analysis was used to compare between groups. (I,J) Representative fluorescent images of Phalloidin-stained (that [file pgen.1011453.s006.tif]

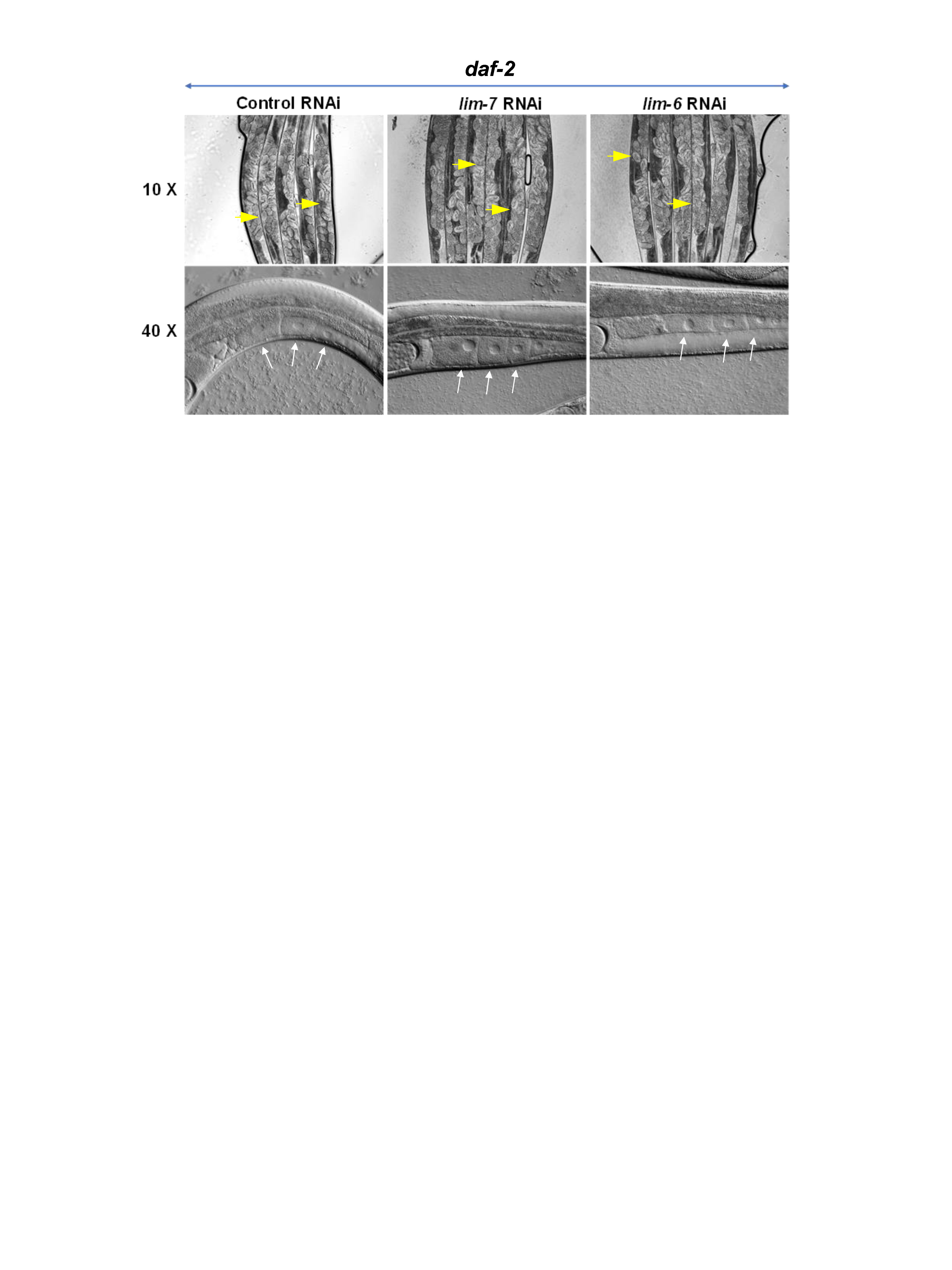

Supplement: S7 Fig — Representative images showing that lim-7 and lim-6 RNAi do not result in sterility in daf-2(e1370) worms. The upper panel shows brightfield images where yellow arrowheads mark eggs. In the DIC images of the lower panel, white arrows point towards the oocytes in the gonadal arm. (TIF) [file pgen.1011453.s007.tif]

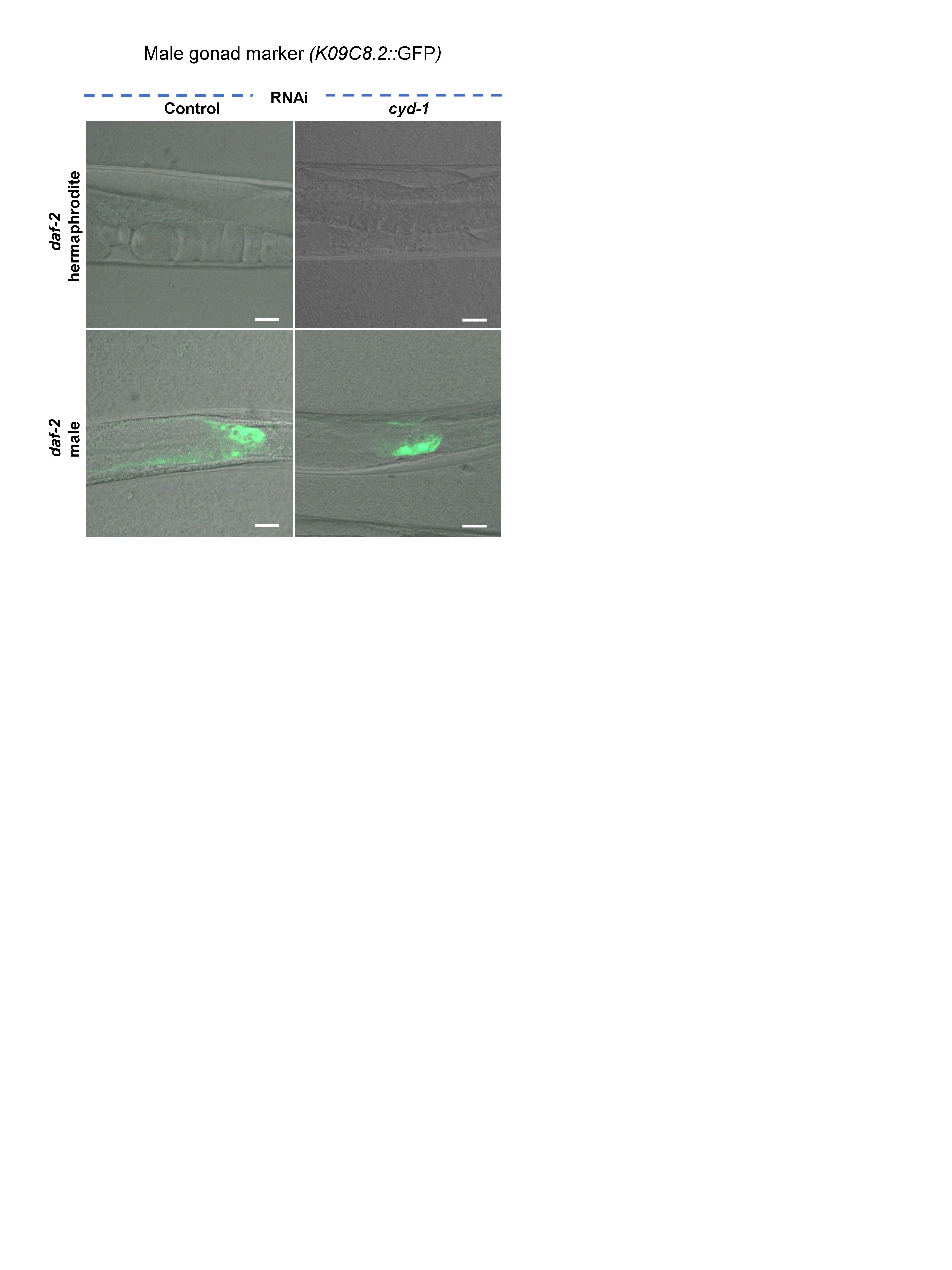

Supplement: S8 Fig — Representative fluorescent and DIC merged images of gonads showing K09C8.2::GFP (male gonad marker) expression in daf-2(e1370) hermaphrodite or male worms (day-1 adult) grown on control and cyd-1 RNAi. Scale bar 20 μm. (TIF) [file pgen.1011453.s008.tif]
