# Supplementary material for: A non-canonical role of somatic Cyclin D/CYD-1 in oogenesis and in maintenance of reproductive fidelity, dependent on the FOXO/DAF-16 activation state
Source: PLoS Genet. 2024 Nov 15;20(11):e1011453. doi: 10.1371/journal.pgen.1011453 (PMC11602045; doi:10.1371/journal.pgen.1011453)
Supplement: Attachment — Submitted filename: point-by-point.pdf [file pgen.1011453.s013.pdf]

Reviewers' comments

Rev. 1:
1. reproductive span - only unmated is shown - what is the mated RS? (1G) Only the mated RS properly measures oocyte quality with age. This should be done as in 1E, F.

Reproductive span for both selfed and mated WT worms is added (Figure 1G).

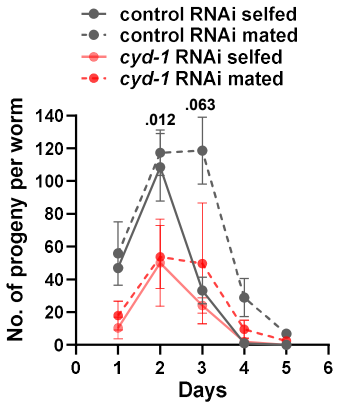

2. imaging is good, non-parametric analyses follow previous established norms – excellent

3. Why was RNAi started so early? what happens when the RNAi starts at the onset of germline (L4) rather than L1? Does the change in endomitotic ooctyes of unc-119 expression still occur if RNAi is started at L4? What phenotypes change?

We did not observe poor oocyte quality or increased endomitotic oocytes upon *cyd-1* KD post-L4 in WT worms. Quantification for the data has been added (Figure S1I,J).

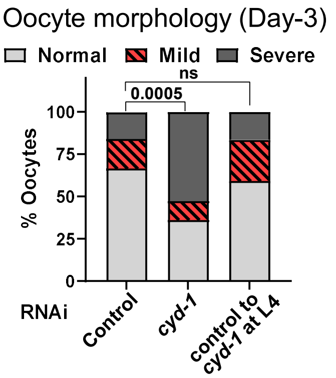

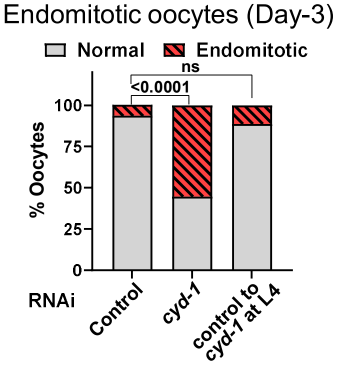

The work is quite sound, with the exceptions noted above. However, the novelty of the observations is limited, and therefore, this work might be better suited to PLoS Genetics with only minor revisions. Specifically, regulation of reproductive aging by IIS and DAF-16, non-autonomous regulation of reproductive aging, including non-autonomous regulation of reproductive aging through the IIS pathway, non-autonomous regulation of reproductive aging by nutrient-sensing pathways, activation of DAF-16 causing developmental delay/slowed reproduction, and regulation of reproductive aging by cell cycle genes have all been shown several times previously, some more than a decade ago. These citations were listed, but the fact that the broader significance of each of the points that the authors make specifically regarding cyclin D and DAF-16 have already been made in other contexts suggest that perhaps the novelty of the findings are not to the level that is appropriate for PLOS Biology.

As rightfully mentioned by the reviewer and also mentioned and cited in the manuscript, previous studies have established:

1. Cell non-autonomous role of IIS/FOXO in oocyte quality maintenance (PMID: 20946987).
2. Other examples of non-autonomous regulation of reproductive aging for example by , TGF beta (PMID: 20946987), CREB (PMID: 32544391).
3. Activated FOXO delays/slows reproduction: PMID: 20946987, PMID: 25960195.

   Regarding FOXO and cyclin D, the following is known:

1. Upon detachment of a cell from the cell matrix, cell death (anoikis) is triggered, where FOXO plays a crucial role. Cyclin D has been shown to inhibit this function of FOXO

in a direct protein-protein interaction, independent of its kinase function, thus promoting tumorigenesis and metastasis in cancer (PMID: 19575018).

2. FOXO3 has been shown to inhibit cyclin D1 expression (PMID: 30064475).

3. Forced expression of FOXO in mouse embryonic fibroblast cell culture, leads to G1 arrest primarily by transcriptional repression of Cyclin D (PMID: 12391153).

Cyclin related to reproductive aging:

1. In mice models, ablation of Nrf2 led to high frequency of chromosome misalignment in oocytes, likely mediated through the Cyclin B1/CDK1 pathway PMID: 30368232

The novelty of our work lies in the following:

1. To our knowledge this is the first study that shows the role of canonical G1-S cyclin, Cyclin D/CYD-1 in regulation of reproductive aging (that too cell non-autonomously, from the uterine tissue).

2. According to this and a recently published study from our lab (PMID: 37577954), perturbation in uterine tissue (DNA damage or in this case cell cycle) can be sensed by the activated DAF-16 which in turn influences the spermatogenesis-to-oogenesis switch (pachytene exit during oogenesis). This underscores the importance of the uterine tissue (egg-laying system) as a central hub for gauging cellular health and cell-intrinsic deficiencies. Previously, DAF-16 in the vulva has been shown to sense the defects in embryo eggshell integrity, thereby modulating maternal proteostasis (PMID: 32217667).
3. Control of the spermatogenesis-to-oogenesis switch dependent on the activation/deactivation status of DAF-16 in response to Cyclin D deficiency is shown for the first time.
4. Contrary to the grand pro-longevity role of FOXO, here we counter-intuitively observe activated FOXO-dependent defects in the somatic gonadal tissues that lead to germline sterility upon cell cycle perturbation in the developing uterine tissue. Very few examples exist where activated FOXO has been shown to have tissue deforming effects (PMID: 28549065).

Significance statement:

The uterus is not a mere bystander or a storage house for eggs before being laid. Rather as we observe in our study, it is capable of influencing oogenesis/oocyte quality, by a yet unknown mechanism. If cell-cycle is perturbed (by Cyclin D RNAi) only in the uterus, reproductive aging is accelerated under normal insulin signalling conditions leading to early decline in oocyte quality. If in such a scenario, DAF-16/FOXO is activated during development (as in under low insulin signalling), it leads to deterioration of somatic gonadal tissues to orchestrate germline arrest leading to sterility with a concomitant increase in levels of immune/defence response (transcription level) genes. Additionally, upon perturbation of somatic gonad development (by *egl-43* or *sys-1* knock-down) activated DAF-16 leads to similar germline arrest, implying DAF-16 can sense deficiencies in the somatic gonad and relay the signal to germline. Thus, here we show a novel role of DAF-16 in monitoring uterine health before reproductive commitment. In the nature, when resources are scarce (under low insulin signalling) this could be an important strategy to minimize energy expenditure and shuttle resources for somatic

maintenance (increase in mRNA levels of immunity or defence response related genes) than produce poor quality oocytes. The communication between uterus and germline can be an interesting arena for future research.

We respect the opinion of the reviewer and suggestions to submit the work to PLoS Genetics. We are sure that the study will appeal to a wide range of scientists.

Rev. 2:
In this manuscript, Rautela et al. identify an interesting new role for cyd-1, encoding the C. elegans cyclin D ortholog, in the development of the germline and somatic reproductive tissues. Notably, cyd-1 interacts with daf-16, the FOXO ortholog, in these roles, producing different phenotypes when daf-16 is active vs. inactive. Furthermore, cyd-1 acts non-cell autonomously from the somatic gonad to regulate germline phenotypes.

Specifically, in this paper the authors show that knocking down cyd-1 activity using RNAi reduces oocyte quality, leading to smaller brood sizes. Indeed, the defects observed include not only endomitosis and misshapen oocytes but partial transdifferentiation of oocytes such that they expressed a neuronal marker. They used tissue-specific RNAi to demonstrate that cyd-1 is required in the somatic gonad (uterus), not the germline, to regulate oocyte quality. They find a daf-16-dependent arrest of germline development at the pachytene stage when cyd-1 RNAi is performed under conditions of activated daf-16 (a daf-2 background). They observed additional defects upon depletion of cyd-1 in the vulva and somatic sheath cells.

In general, the experiments were carefully done, including several controls that I really appreciated. For example, the controls to ensure that the tissue-specific RNAi was truly specific to the tissues of interest greatly increased confidence in those results. In addition, confirmation by qPCR of the relative efficiency of RNAi in daf-2 vs. daf-16; daf-2 was very helpful for interpreting those experiments.

This paper, with suggested edits below, would be of interest to scientists studying germline development, oocyte quality, cyclins, and FOXO pathways. Below I provide additional comments intended to strengthen this paper.

Major comments
1. One major disconnect I had when reading this paper was the lack of any connection made between cyd-1 and cdk-12. In a 2023 paper published by this lab, they described a role for cdk-12 that seems very analogous to what they describe for cyd-1 in the current work. Specifically, they showed that RNAi of cdk-12 in daf-2 mutants causes a daf-16-dependent germ cell arrest in the pachytene stage. They found the focus of cdk-12 activity to be the uterus, within the somatic gonad. In a wild-type background, cdk-12 RNAi decreased oocyte quality. All of these very specific phenotypes also occur upon RNAi of cyd-1, as described in this paper.

At the start of this article, the authors explain that because the canonical cyclin that binds CDK-12 (cyclin K) did not appear to play a role with respect to these phenotypes, they screened additional cyclins for roles in oogenesis, thereby discovering cyd-1. This implied to me that CYD-1 might bind to CDK-12 in this context, which would be different than the known binding partners for these proteins and would be an interesting finding. Even if these proteins are not direct binding partners, the very similar phenotypes imply that there could be some similar mechanism at play or other genetic interactions occurring. However, cdk-12

was not mentioned again in the paper, even as the same phenotypes observed with cdk-12 RNAi were uncovered for cyd-1. Indeed, the term "surprising" was used when these similar phenotypes were described, when to me the phenotypes seemed somewhat expected, given what was known about cdk-12.

To me, the potential relationship between cyd-1 and cyk-12 should be addressed in this paper, whether that be in writing or with additional experiments. The authors can decide how important it is to test these ideas experimentally, but failing to mention a possible connection seems like an omission.

The sterility phenotype of *daf-2* is similar for both *cdk-12* and *cyd-1* KD. However, while *cdk-12* KD led to DNA damage, we found that CYD-1 is not involved in DNA damage response (DDR). This is because the wild-type late-L4 staged worms grown on control or *cyd-1* RNAi showed no down-regulation of DDR genes (**Figure S3F**) that we typically observed in case of *cdk-12* KD (PMID: 37577954). Additionally, exposure of WT worms to gamma radiation led to a similar percentage of eggs hatching for both control and *cyd-1* KD groups (**Figure S3G**). Unlike *cdk-12* KD, *cyd-1* KD led to the deterioration of somatic gonad in *daf-2* worms (**Figure 4F,G**; **5D**). Therefore, although phenotypically *cdk-12* and *cyd-1* KD show similar sterility under low insulin signaling (*daf-2* worms), the underlying mechanisms differ. These data are added in text on page 14, line 24-31.

The newly added data is as below:

mRNA fold change
DNA damage Response (DDR) genes

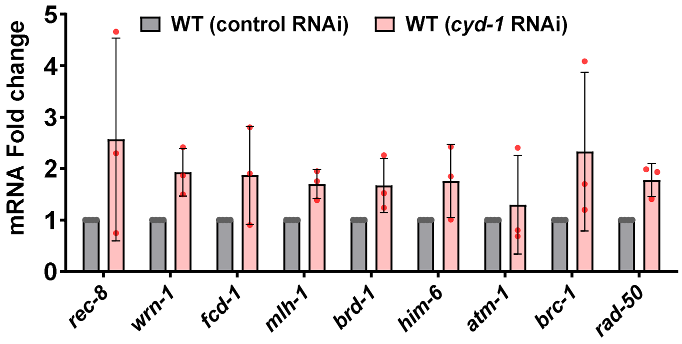

Percent egg hatching

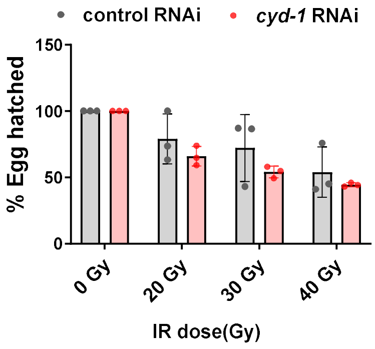

2. Statistics: Welch's t-tests or ANOVAs are used throughout this paper for all experiments. T-tests are useful for parametric (normally distributed), continuous data. However, some experiments use categorical data, where, in contrast to continuous data, the output of the experiment is assigning worms to a category, for example, "Normal", "Mild", or "Severe". In these cases, a t-test cannot be used because the only numbers that can be entered to analyze are the percent of worms within a category. This analysis does not take into account the numbers of animals used to determine the means that are compared. For example, consider a case where the mean of a control group is 33% and the mean of an experimental group is 50%. If the actual data that went into those means was 1 worm out of 3 (control) vs. 2 worms out of 4 (experimental), intuitively we might guess that those differences are likely due to chance. By contrast, if the differences were 33 worms out of 100 vs. 50 worms out of 100, those differences might be more meaningful. With more numbers, the differences become even more convincing. With a t-test there is no way to distinguish between those

two cases. Using a statistical test designed for categorical data seems to me to be more appropriate for these experiments.
(See https://www.ncbi.nlm.nih.gov/pmc/articles/PMC4966396/ for one source of information about these tests).

We agree with the reviewer. Chi square test is also used in the following papers (PMID: 38370685, PMID: 29478855) for similar experiments. We have modified the statistics throughout the paper and used chi-square test for categorical data.

3. I did not understand why RNA-seq data were performed using worms where cyd-1 knockdown occurred in the germline only, when the authors showed that the oogenesis phenotypes were caused by knockdown in the somatic gonad. Please explain how the RNA-seq data should be interpreted in light of the focus of cyd-1 action.

We apologise for the confusion. The transcriptomics was performed with KD only in the somatic gonad (egg laying apparatus only) and not in the germline. For this, the unc-62 promoter driven *rde-1* line was used (CGC strain name: NK741). (PMID: 24780738). We have clarified this in the manuscript (Page 23, Lines 3-5)

Minor points
1. I appreciated the table with the list of strains and gen.otypes (p. 34), which is very helpful. However, in some cases the information about the transgenes was not fully clear. Can information be added to the table when necessary to clarify which lines are single copy/multicopy, extrachromosomal/integrated? For example, strain 4 CU1546 is listed as ced-1p::ced-1::GFP, which does not include the transgene name. The transgene name should provide the necessary information. Similarly, please update any non-standard nomenclature. For example, what do capital letters before a number signify? (e.g., WM27 in strain 1 generated in-house). Additionally, what does "(genomic)" mean? Finally, if any transgenes were generated for this study, please provide details on how the strain was made. (I wasn't sure if any of the "in-house" strains included making new transgenes). WormBase has details on nomenclature
(https://wormbase.org/about/userguide/nomenclature).

We have added the transgene names and updated our strain list accordingly.

2. For stacked bar graphs with multiple categories, only the error bars for the top category are easily visible. I did not realize at first that each category had its own error bars. Can the formatting be adjusted to make the other error bars more visible?

For stacked bars, we have now modified the statistics and used chi-square test where no error bars are plotted.

3. Fig. S2C, S2I. I cannot see the eggs on the plates. Can the images be larger?

The image has been zoomed to make it more visible.

S2C:

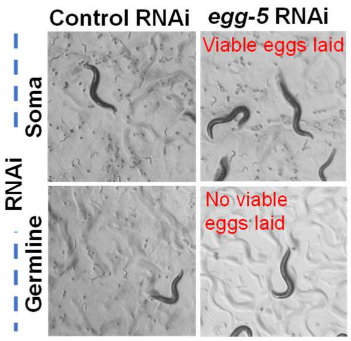

S2I:

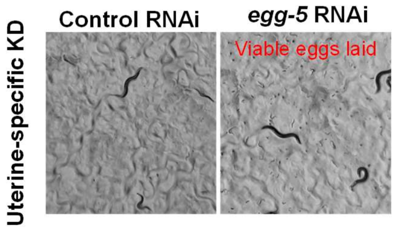

4. p. 13 line 21, there is a reference to data not shown. Why not show these data in the supplement?

We have added the SV314 (*cyd-1(he112)/+*) data for oocyte quality (**Figure S3B,C**) and endomitotic oocytes (**Figure S3D,E**).

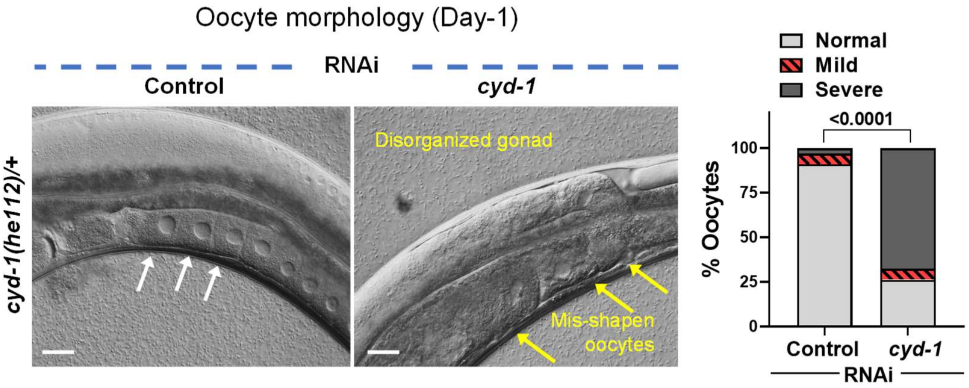

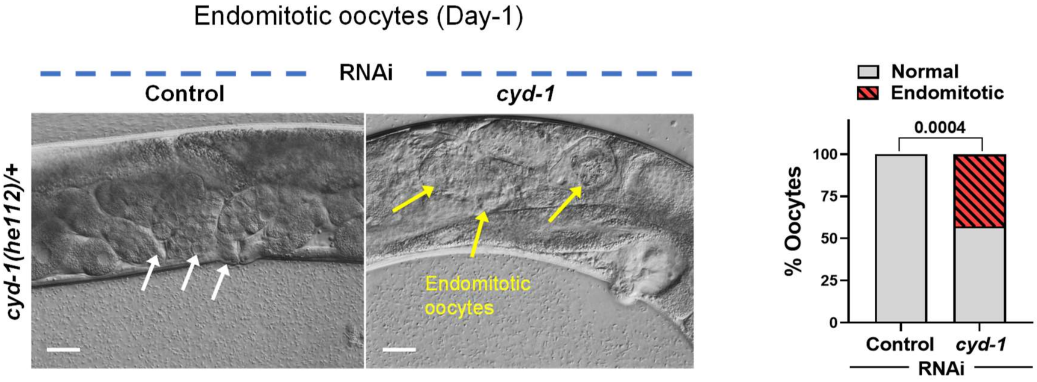

5. Figure S4A. Please include information in the figure legend explaining which promoters were used to drive tissue-specific RNAi for this experiment.

The Figure S4A of the earlier version is Figure S5A in the current version of the manuscript. Details of the promoter has been added to the legend. The following promoters were used to drive the tissue-specific expression of *rde-1* (vulva: *lin-31*; DTC: *lag-2*; hypodermis: *lin-26*;

muscle: *hlh-1*; intestine: *nhx-2)* in a *rde-1(ne219) mutant.* For neuron-specific RNAi, *unc-119p* was used to drive the expression of *sid-1* cDNA in the neurons of *sid-1(pk321)* mutant. Page 13 of supplementary file, line no. 2-7.

6. p. 17. The terminology used to describe the vulval phenotypes does not appear to fully match the experiments shown. There is a statement that the number of vulval cells is reduced, but what is shown is reduced/aberrant expression of AJM-1::GFP, with no quantification of vulval cell number. The AJM-1::GFP expression defect could be due to various causes, not just number of cells. Furthermore, the expression of cdh-3::gfp is used to describe a "VPC fate" defect. However, VPC fate is best observed in the L3 stage, prior to cdh-3::gfp expression, and typically uses markers for 1˚ or 2˚ fate. It is not clear that abnormal cdh-3::gfp expression is occurring because of a loss of VPC fate. While that is a possibility, it could also be a problem with differentiation or morphogenesis. Additionally, the "abnormal expression" appears from Fig 4H to include a loss of cdh-3::gfp in the uterine cells. I suggest modifying the language of this section so that the phenotypes described more accurately represent the data shown.

AJM-1::GFP was utilized to look at the vulval structure not the cell division. The vulval cells expected to express *cdh-3::gfp* do not do so in the absence of *cyd-1*, but as rightly mentioned by the reviewer 2, it could be a problem in cell division or acquiring appropriate vulval cell fate. We have modified the language as below:

“Next, we asked if these defects in vulva were due to impairment in the vulva development (vulval cell divisions or cell fate acquisition). We observed the vulval precursor cell (VPC) fate of late-L3 staged worms using the transgenic reporter strain, *cdh-3::gfp* (Pettitt et al., 1996). In *daf-2* worms fed control RNAi, we observed the correct expression of *cdh-3::gfp* in vulval cells, however, the expression was abnormal or absent upon *cyd-1* RNAi, suggesting incomplete or aberrant vulval development.” We have modified the text in Page No. 18, Line no. 30 to Page No. 19, Line No. 2.

7. Page 27 lines 11-13 state that sperm number did not increase on day 3, but Fig 6C seems to show many more sperm in cyd-1 RNAi vs. control.

We have replaced the control RNAi image.

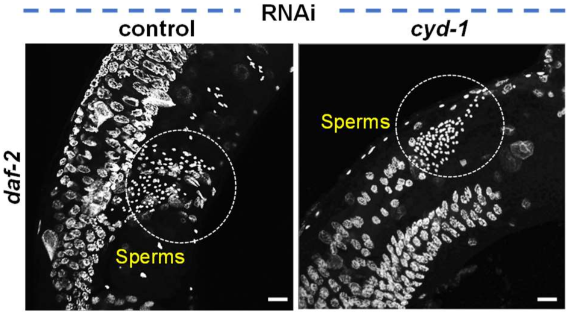

Rev. 3:
The authors characterize in this study the effect of cyclin cyd-1 knock-down on the reproductive system in wild-type and insulin-IGF-1 signaling mutant C. elegans. Cyd-1 is a G1-S phase cyclin and knock-down in uterine cells reduces oocyte number, quality, and reproductive span. In daf-2 mutants, additional knock-down of cyd-1 in the gonads induces reduction of germ cells in the transition as well as pachytene zone. These double mutant

animals do not produce mature oocytes and are infertile. Further analysis revealed morphological defects of the somatic gonad tissue and the egg-laying apparatus. This study is in general well-done with well-designed figures. The authors investigate the interesting aspect of non-classical function of cyclins during reproduction. However, it is unclear whether the observation reveal underlying developmental defects or accelerated aging phenotypes.

Major comments:
1) The authors compare the morphology and organization of the gonad as well as oocyte quality of 3-day old animals with and without cyd-1 knock-down. They interpret the observed effects as reproductive senescence. Is it possible that these effects are caused during development since the knock-down started in the early L1 larval stage? Do the authors see similar phenotypes if they knock-down cyd-1 only in adult animals (wild-type, daf-2, and daf16 daf-2)? To clearly show this accelerated reproductive senescence phenotype, it would be helpful to have a figure that allows the direct comparison of wild-type and daf-2 C. elegans gonads with and without cyd-1 knockdown from the first day of adulthood until wild-type animals show age-related changes of the gonads. In addition, longitudinal egg-laying curves would help to clarify whether cyd-1 knock-down only accelerates age-related changes or whether the egg number is reduced even in young age. Since daf-2 mutant animals exhibit a delay in their age-related changes- how does this affect the phenotypes observed after cyd-1 knock down? It is also unclear whether the reproductive spans in Figure 1G are measured in mated or in self-fertile animals that are sperm depleted.

The poor oocyte quality observed in WT worms with *cyd-1* KD is not apparent if *cyd-1* is KD post development (L4 onwards) (New figure added as **Figure S1I,J**), nor do *daf-2* worms arrest if *cyd-1* is KD post development, implying the essential role of *cyd-1* during early development that influences reproductive aging in WT and oogenesis in *daf-2*. The reproductive span in **Figure 1G** has been updated to show both selfed and mated reproductive span. Since *daf-2* worms arrest upon *cyd-1* KD, we have not done reproductive span for *daf-2* worms.

2) The authors describe changes in vulva morphology as well as sheath cell structure after knockdown of cyd-1 in daf-2 mutant animals. Is this phenotype completely absent after cyd-1 knockdown in wild-type animals?

The vulva morphology and sheath cell structural defects (Figure S6I,J) were visibly absent in WT *cyd-1* depleted worms. However, in day-3 adult gonads, we do see a reduction in *lim-7p::gfp* expression (Figure 5F) upon *cyd-1* KD.

3) The authors refer on page 13 and page 22 to data sets that are not shown. These data sets need to be included.

We have now added the data in Page 13. However, due to space constraints in the main figure or supplementary figure, we have not added that data on the page 22. We have shown that data below. If the reviewer insists, then we would add the data and reshuffle all the figures.

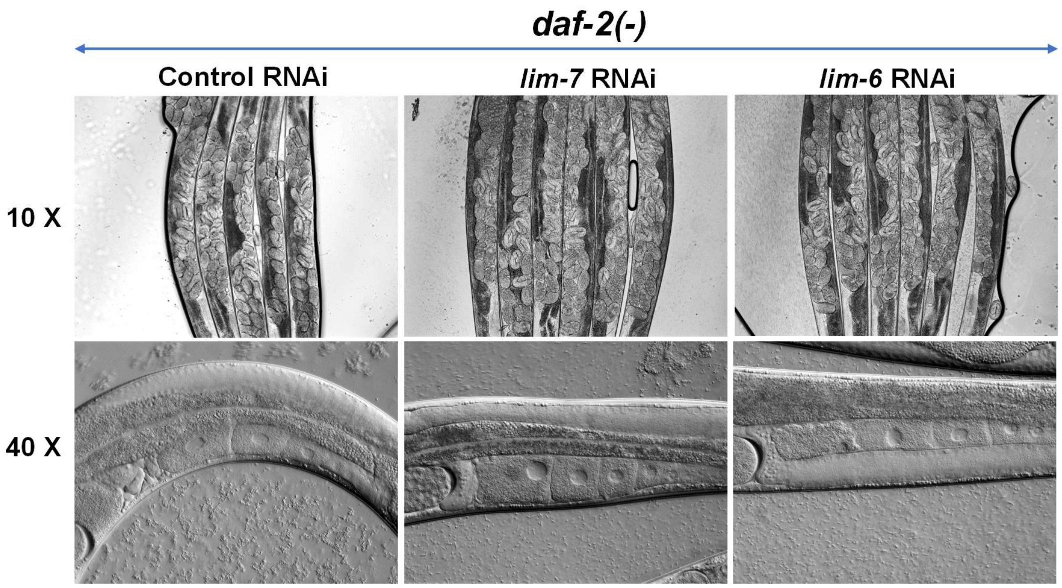

4) The first paragraph of the results section is confusing. It seems that the cye-1 and cyd-1 knock-outs started the project, however, starting with these data sets and ending the first paragraph with the statement that cyd-1 knockouts are not useable within the context of this paper is not helpful and limits the readability of the study. Restructuring the results section and discussing the knockouts in a different part of the paper will strengthen the paper.

The study started by screening for sterility in *daf-2* worms, by knocking down the cyclins using RNAi and not by using mutants. The mutants of these cyclins are lethal and arrest at larval stages, making it impossible to use them for the study. So, we resorted to the use of RNAi.

5) The subfigures of Figure S2C and I that show the overview need better labeling. What do the authors exactly try to show? The yellow labeling of viable eggs or not viable eggs is confusing since it is impossible to see eggs in these overviews. A more detailed figure legend or an additional panel showing the quality of the laid eggs would be helpful.

We show that KD of *egg-5* leads to dead eggs (no viable progeny) in germline-specific RNAi but shows viable progeny in soma-specific or uterine specific RNAi. We have modified the figure and the text.

6) It would be helpful to show standard deviation instead of the standard error of the mean.

We have changed standard error of the mean to standard deviation.

Minor comments:
1) The order in Figure S5 is confusing.

We have changed the way the figure is labelled.

2) The meaning of the color and size of the dots in figure S5A,B is unclear.

Both the color, as well as the dots signify the “adjusted p-value’’. The bigger the dot is (or more yellow) it means, it has more significant p-value than the smaller, blue dots.

3) Page 7 line 9 delete "the"

Noted.
