# Supplementary material for: A non-canonical role of somatic Cyclin D/CYD-1 in oogenesis and in maintenance of reproductive fidelity, dependent on the FOXO/DAF-16 activation state
Source: PLoS Genet. 2024 Nov 15;20(11):e1011453. doi: 10.1371/journal.pgen.1011453 (PMC11602045; doi:10.1371/journal.pgen.1011453)
Supplement: Attachment — Submitted filename: Response to the reviewers (REV2).docx [file pgen.1011453.s014.docx]

Reviewer #1: All of my prior comments have been carefully addressed. I find no outstanding issues to correct.

**Reply:** We thank the reviewer for the constructive reviews that helped improve the manuscript.

Reviewer #2: The authors made an effort to address most of my concerns. However, they reinforced my concern about their interpretation that the observed effects show reproductive senescence. The additional data provided confirms that the observed oocyte defects are rather due to developmental defects than premature aging. cyd-1 RNAi in L4 does not reduce oocyte quality. Animals exposed to cyd-1 RNAi in L1 exhibit lower overall egg production and do not stop egg laying premature. Furthermore, these animals show never a “young phenotype”- quite the contrary, all results point to developmental defects that lead to reproductive defects that get worse with age- but "sick" getting "sicker" is not a reproductive aging phenotype.

I also want to point out that data that is discussed in the paper needs to be included for the reader. So, I "insist" to include the data from former page 13 as wells page 22 (now page 23 "We inquired if knocking down genes essential for sheath cell development (e.g., 23 lim-7, lim-6) would also lead to germline arrest in daf-2 but found no such sterility (data not shown).") that was not shown in the first version of the manuscript.

**Reply:** We thank the reviewer for the constructive review that improved the manuscript. We agree with the reviewer. We have now textually modified the title as well as the text of the manuscript to not claim the effect on reproductive aging following CYD-1 knockdown in wild-type.

All data that are referred to in the manuscript are now presented. The data that was not shown before has now been added as Figure S7.

Reviewer #3: I agree with the previous reviewer 1's comments that the additional data provided support a developmental role for cyd-1 in oogenesis, and not reproductive aging (at day 1 the fertility of cyd-1 RNAi is already lower than control). I suggest the authors to tone down their conclusions to avoid any suggestions on a reproductive aging phenotype.

**Reply:** We thank the reviewer for the comments, and we have modified the title as well as the text to avoid claiming regulation of reproductive aging by CYD-1.

**Have all data underlying the figures and results presented in the manuscript been provided?**
Large-scale datasets should be made available via a public repository as described in the *PLOS Genetics* [data availability policy](http://c05y1x9s.r.us-east-2.awstrack.me/L0/http:%2F%2Fjournals.plos.org%2Fplosgenetics%2Fs%2Fdata-availability/2/010f01917d1ffe32-b5c082ed-a740-4dec-8c98-f259e34a1a97-000000/-fBMSysbN3AEY2IhGC0xyOrqLRo=173), and numerical data that underlies graphs or summary statistics should be provided in spreadsheet form as supporting information.

Reviewer #1: Yes

Reviewer #2: **No:** I asked in my first review to include data sets on page 13 and page 22 (now page 23 "We inquired if knocking down genes essential for sheath cell development (e.g., 23 lim-7, lim-6) would also lead to germline arrest in daf-2 but found no such sterility (data not shown).") that are not shown.
They added the data from page 13 in the revised manuscript, but only showed the data from page 22 in their response to the reviewers. These need to be added to the manuscript.

**Reply:** We have now added the data in the new Figure S7.

Reviewer #3: Yes
